# The College of Surgeons and Physicians of the University of the State of New-York

**Published:** 1837

**Authors:** 


### Art VI.—The College of Surgeons and Physicians, of the University of the State of New York.

It appears that the Regents have commenced the work of *reform* by the election from the West, of a Professor of Surgery, in place of Dr. Stevens. We have often been astonished, in latter years, that the College should have such small classes ; but the cause is at length disclosed. It was the ignorance of its professors. What could be expected of incumbents *inferior to the new appointee ?*—a smatterer in Anatomy —in Surgery a mechanic ; a man whose fondest friends have not claimed for him either talents or science ; who is profoundly ignorant of the grammar and orthography of his mother tongue ; who is *not a graduate*, and could not, *on examination*, receive a degree, *even* from the college from which he was taken, or in which he has been placed ; who was elected into the former, on a recommendation designed as a hoax ; who could not, and did not, sustain himself, even in *that* institution ; who has been three times published as a liar in Cincinnati ; and who left it without disclosing to his colleagues or the Trustees of the College to which he belonged, that he was about to decamp. It has been reported, *mirabile dictu*, that he had been *sent for* by the good people of New York, to supply the places of Mott and Bushe ! Verily, to borrow an idea from Peter Pindar, medical skill must have been at a low ebb in that city, when they were driven to make *such* an importation. But New York is an importing city, and may sometimes mistake *brass* for gold. Major Noah suggests that none of *her native* surgeons might have been willing to relinquish a lucrative practice, for the purpose of taking a professor's chair in her College—implying a suspicion, that the new incumbent may not have been called upon to make such a sacrifice. He could have his suspicions converted into convictions, by enquiring of the good people of Danville, Louisville, and Cincinnati. But why should an appointment into the New York school destroy a man's practice ? Only, it would seem, by degrading him. Of this sinister effect, the new surgical professor need feel no apprehensions. When the facetious Major, a few years since, in personating the Empire State, spoke of putting little Delaware into his breeches pocket, he could not have anticipated, that in 1837, one of her sons would have been called to the premiership of a university, with which, in the American mind, are associated the venerated names of Bard, Mitchell, Miller, Post, Hosack, McNeven, Francis, and Mott, *cum multis aliis*, of like dignity and reputation. We trust the Major will have the magnanimity to take back his sarcasm, *before* the opening of the College; and henceforward speak of little Delaware with becoming respect. We have not the honor of knowing the Regents of the University of New York, and can, therefore, commit no personality, when we doubt whether a man's having wandered for a few years from town to town, in the West, the "laughing stock" of the educated profession, will fit him for a professorship in their school—low as they have degraded it; but if they think otherwise, then let them elect Dr. Williams, the *Oculist.* He would probably *open their eyes* to their true position. It would be absurd for us not to admit the general superiority of the West, but powerful as are its influences, they cannot transmute eastern dunces into learned philosophers. D.

